# Nesfatin-1 Stimulates CCL2-dependent Monocyte Migration And M1 Macrophage Polarization: Implications For Rheumatoid Arthritis Therapy

**DOI:** 10.7150/ijbs.77987

**Published:** 2023-01-01

**Authors:** Jun-Way Chang, Shan-Chi Liu, Yen-You Lin, Xiu-Yuan He, Yi-Syuan Wu, Chen-Ming Su, Chun-Hao Tsai, Hsien-Te Chen, Yi-Ching Fong, Sung-Lin Hu, Chien-Chung Huang, Chih-Hsin Tang

**Affiliations:** 1 The Ph.D. Program of Biotechnology and Biomedical Industry, China Medical University, Taichung, Taiwan.; 2 Department of Medical Education and Research, China Medical University Beigang Hospital, Yunlin, Taiwan; 3 School of Medicine, China Medical University, Taichung, Taiwan; 4 Department of Sports Medicine, College of Health Care, China Medical University, Taichung, Taiwan; 5 Department of Orthopedic Surgery, China Medical University Hospital, Taichung, Taiwan.; 6 Department of Orthopedic Surgery, China Medical University Beigang Hospital, Yunlin, Taiwan; 7 Department of Family Medicine, China Medical University Hsinchu Hospital, Hsinchu, Taiwan; 8 Division of Immunology and Rheumatology, Department of Internal Medicine, China Medical University Hospital, Taichung, Taiwan.; 9 Graduate Institute of Biomedical Sciences, China Medical University, Taichung, Taiwan; 10 Chinese Medicine Research Center, China Medical University, Taichung, Taiwan; 11 Department of Biotechnology, College of Health Science, Asia University, Taichung, Taiwan

**Keywords:** rheumatoid arthritis, nesfatin-1, CCL2, M1 macrophages, synovial fibroblasts

## Abstract

Rheumatoid arthritis (RA) is a prototypic inflammatory disease, characterized by the infiltration of proinflammatory cytokines into the joint synovium and the migration of mononuclear cells into inflammatory sites. The adipokine nesfatin-1 is linked to inflammatory events in various diseases, although its role in RA pathology is uncertain. Analysis of the Gene Expression Omnibus GSE55235 dataset revealed high levels of expression of the adipokine nesfatin-1 in human RA synovial tissue. Similarly, our human synovial tissue samples exhibited increasing levels of nesfatin-1 expression and *Ccl2* mRNA expression. Nesfatin-1-induced stimulation of CCL2 expression and monocyte migration involved the MEK/ERK, p38, and NF-κB signaling pathways. Notably, nesfatin-1-induced increases in CCL2 expression favored M1 macrophage polarization, which increased the expression of proinflammatory cytokines IL-1β, IL-6, and TNF-α. Finally, nesfatin-1 shRNA ameliorated the severity of inflammatory disease and reduced levels of M1 macrophage expression in CIA mice. Our studies confirm that nesfatin-1 appears to be worth targeting in RA treatment.

## Introduction

Rheumatoid arthritis (RA) is an autoimmune inflammatory disease associated with irreversible destruction of the affected joints 1, 2. The trigger that causes RA-related immune system dysfunction remains to be elucidated 3. Synovial inflammation and immune cell infiltration are common features of RA synovium pathology 4, 5. In RA, activated synovial fibroblasts (RASFs) produce proinflammatory cytokines and chemokines that contribute to the characteristic joint inflammation and cartilage destruction. At the sites of inflammation, the inflammatory chemokines recruit monocytes, neutrophils and various cell types that promote the inflammation, while other types of cells such as leukocytes can produce inflammatory cytokines, resulting in the so-called “vicious cycle” of disease activity in the RA synovial microenvironment 6.

The migration and infiltration of monocytes to lesions of inflammatory is mediated by inflammatory/inducible cytokines that are preferentially produced in peripheral tissue at those sites; c-c motif chemokine ligand 2 (CCL2, also known as monocyte chemoattractant protein-1 [MCP-1]) is particularly important for its chemotactic activities in RA 7. CCL2 provokes the recruitment and locomotion of monocytes to inflammatory sites in numerous inflammatory diseases such as arthritis, atherosclerosis, and cancers 8, 9. RA is characterized by high levels of CCL2 production in the serum, synovial fluid, and synovial tissue 10-12. CCL2 promotes the development of synovitis in the inflamed human knee 13 and increases macrophage infiltration into rabbit joints injected with CCL2 14.

After migrating to the inflammatory sites, monocytes differentiate into macrophages and become major contributors to synovial inflammation 15, with evidence showing that synovial macrophages are linked to RA severity and cartilage destruction 16. In the immune microenvironment, diverse stimuli transform naïve macrophages into the proinflammatory M1 or anti-inflammatory M2 phenotypes 17. The M1 phenotype expresses various proinflammatory cytokines, including interleukin (IL)-1β, IL-6 and tumor necrosis factor (TNF)-α, while M2 phenotypes express cytokines that are linked to tissue remodeling, including transforming growth factor-β (TGF-β), IL-10 and arginase 1 (Arg-1) 18. M1 macrophages predominate in RA synovial tissue 19. Thus, synovial cytokines produced by M1 macrophages are important inflammatory factors in RA progression 19.

The release of adipokines from adipose tissue is key to the inflammatory and immune responses in rheumatoid diseases 20. Several adipokines are implicated as risk factors of RA 20, including nesfatin-1. Nesfatin-1 is an 82-amino acid polypeptide that originates from its precursor NUCB2, and was the first time to be discovered in hypothalamic neurons 21. Recent investigations have identified that nesfatin-1 participates in many different diseases and that this neuropeptide is a serum marker for RA 22. Levels of nesfatin-1 expression in human RA synovium and synovial fluid correlate with RA severity 23. Our analysis of human synovial tissue from the Gene Expression Omnibus (GEO) database records revealed high levels of nesfatin-1 expression in RA patients compared with healthy individuals. However, the detailed mechanisms of nesfatin-1-induced inflammation in RA pathology remain undefined.

In this study, we discovered that nesfatin-1 stimulates CCL2 expression via the MEK/ERK, p38 and nuclear factor-κB (NF-κB) signaling pathways. Subsequently, CCL2 promotes monocyte migration and M1 macrophage polarization, critical events in RA disease progression. These results provide new insights into how the adipokine nesfatin-1 worsens RA pathology by increasing CCL2 expression.

## Materials and Methods

### Immunohistochemical (IHC) staining

Human synovial tissues and ankle joints from CIA mice were stained with nesfatin-1 or CCL2 antibody (1:200), then photographed and analyzed using TissueFAXS Spectra (Vienna, Austria). CIA and control joints were counterstained with hematoxylin/eosin (H&E) and Safranin O/Fast Green, then photographed under a light microscope for histological changes and evidence of bone erosion. The histopathological analysis of cartilage destruction and inflammation scores was evaluated blinded by researchers according to previous studies 24.

### Measurements of nesfatin-1 and CCL2 production by enzyme-linked immunosorbent assay (ELISA)

In order to measure nesfatin-1 and CCL2 production, the levels of nesfatin-1 and CCL2 expression in synovial fluid were assayed by the human nesfatin-1 and human CCL2 ELISA kit, according to the manufacturer's protocols. The detailed source of ELISA kits is listed in Supplementary Table S1.

### Western blot analysis

Cell lysates were subjected to SDS-PAGE then transferred to PVDF membranes. The membranes were blocked with 5% non-fat milk, then treated with primary antibodies After washing and treating the membranes with corresponding HRP-conjugated secondary antibodies, protein expression was photographed by an Image Quant LAS 4000 camera (GE Healthcare Life Sciences), as according to previous protocols 25. The detailed source of antibodies is listed in Supplementary Table S2.

### Cell culture

The human RASF cell line MH7A was purchased from Riken (Ibaraki, Japan) and the human acute monocytic leukemia THP-1 cell line was obtained from the American Type Culture Collection (Manassas, VA, USA). MH7A and THP-1 cells were cultured in RPMI-1640 medium contained 10% FBS, penicillin and streptomycin at 37°C under a humidified atmosphere of 5% CO_2_.

### Quantification of mRNA by real-time quantitative polymerase chain reaction (qPCR)

Total RNA was extracted from the MH7A and THP-1 cells using a TRIzol™ kit. Complementary DNA was synthesized from 1 μg of total RNA using an MMLV RT kit (Invitrogen). Analysis of qPCR was carried out with the SYBR™ Fast SYBR™ Green Master Mix (Applied Biosystems, CA, USA). Gene mRNA expression was examined by the StepOnePlus™ Real-Time PCR System, with β-Actin served as the internal control. qPCR primers are listed in Supplementary Table S3.

### *In vitro* chemotaxis assay

All THP-1 migration assays were performed using Transwell^®^ 24-well plates. MH7A cells were pretreated with the p38, ERK, JNK, MEK and κB inhibitors for 30 min, or transfected with their respective small interfering RNAs (siRNAs) for 16-18 h; then treated with nesfatin-1 (Peprotech, USA) for another 24 h. THP-1 cells (1.5 × 10^4^ in 200 μl RPMI medium) were seeded in the upper chamber, and 300 μl of the conditioned medium (CM) from nesfatin-1 treated MH7A cells was placed in the lower chamber. Cells on the underside of the Transwell^®^ membrane were counted and examined under a microscope. The detailed source of inhibitors and siRNAs are listed in Supplementary Table S4-S5.

### THP-1 polarization assay

THP-1 cells were prepared in 6-well plates (1 × 10^6^ cells/well) and cultured in RPMI medium supplemented with 10% FBS, then incubated with phorbol 12-myristate 13-acetate (PMA) (Sigma- Aldrich; P8139; 100 ng/ml) for 24 h to enable differentiation into naïve (M0) macrophages, then incubated in RPMI medium for 48 h. Cells were washed with phosphate-buffered saline (PBS), then with 24 h of treatment with interferon (IFN)-γ (Peprotech; 300-02; 20 ng/ml) and lipopolysaccharide (LPS) (Sigma- Aldrich; L2630; 100 ng/ml) to polarize into M1 macrophages, or treatment with IL-4 (Peprotech; 200-04; 20 ng/ml) and IL-13 (Peprotech; 200-13; 20 ng/ml) to polarize into M2 macrophages. To examine the effect of nesfatin-1 on M1 polarization, the M0 macrophages were pretreated with nesfatin-1-treated MH7A CM and maintained in a ratio of 1:1 serum-free medium for further analysis.

### NF-κB luciferase assay

NF-κB luciferase plasmid (Stratagene; St. Louis, MO, USA) were transfected into MH7A cells using Lipofectamine 2000, follow by treatment with pharmacological inhibitors and nesfatin-1. The Dual-luciferase^®^ Reporter Assay System was used to examine luciferase activity, as according to our previous procedure 26.

### Chromatin immunoprecipitation (ChIP) assay

The ChIP assay was conducted according to the protocol described in our previous report 26. The forward primer, 5'-CCTGGAAATCCACAGGATGC-3' and a reverse primer, 5'-CGAGAGTGCGAGCTTCAG-3', which were subjected to amplify across the CCL2 promoter region (-209 to -9) contain p65 binding site by qPCR assay.

### Immunofluorescence staining

Human synovial tissue samples and CIA mouse ankle and phalanges sections were stained with primary CD68 (1:200), CD86 (1:200) and F4/80 (1:200) antibodies overnight, then incubated with secondary antibodies for 1 h followed by 4',6-diamidino-2-phenylindole (DAPI) for 5 min at room temperature. Images were analyzed with TissueFAXS Spectra (Vienna, Austria). THP-1 cells were seeded on 12 mm coverslips in 24**-**well plates then incubated with nesfatin-1-treated MH7A CM, as described previously 27. The cells were stained with primary CD68 (1:200) and CD86 (1:200) antibodies, then with secondary antibodies and DAPI. Images were captured under a fluorescence microscope (Zeiss; Berlin, Germany).

### Statistical analysis

All statistical analyses were carried out using GraphPad Prism 8.0 (GraphPad software) and all values are expressed as the means ± standard deviation (SD). The Student's *t*-test or one-way ANOVA followed by Newman-Keuls post hoc test were used to compare the differences between the experimental groups. A *p*-value of <0.05 was considered to be significant.

For detailed methods regarding the analysis of the GEO database, human clinical samples, the collagen-induced arthritis (CIA) mouse model, and micro-computed tomography (μ-CT), please refer to the Supplementary Methods.

## Results

### Nesfatin-1 is highly expressed in human RA and CIA synovial tissue

**Figure 1:**
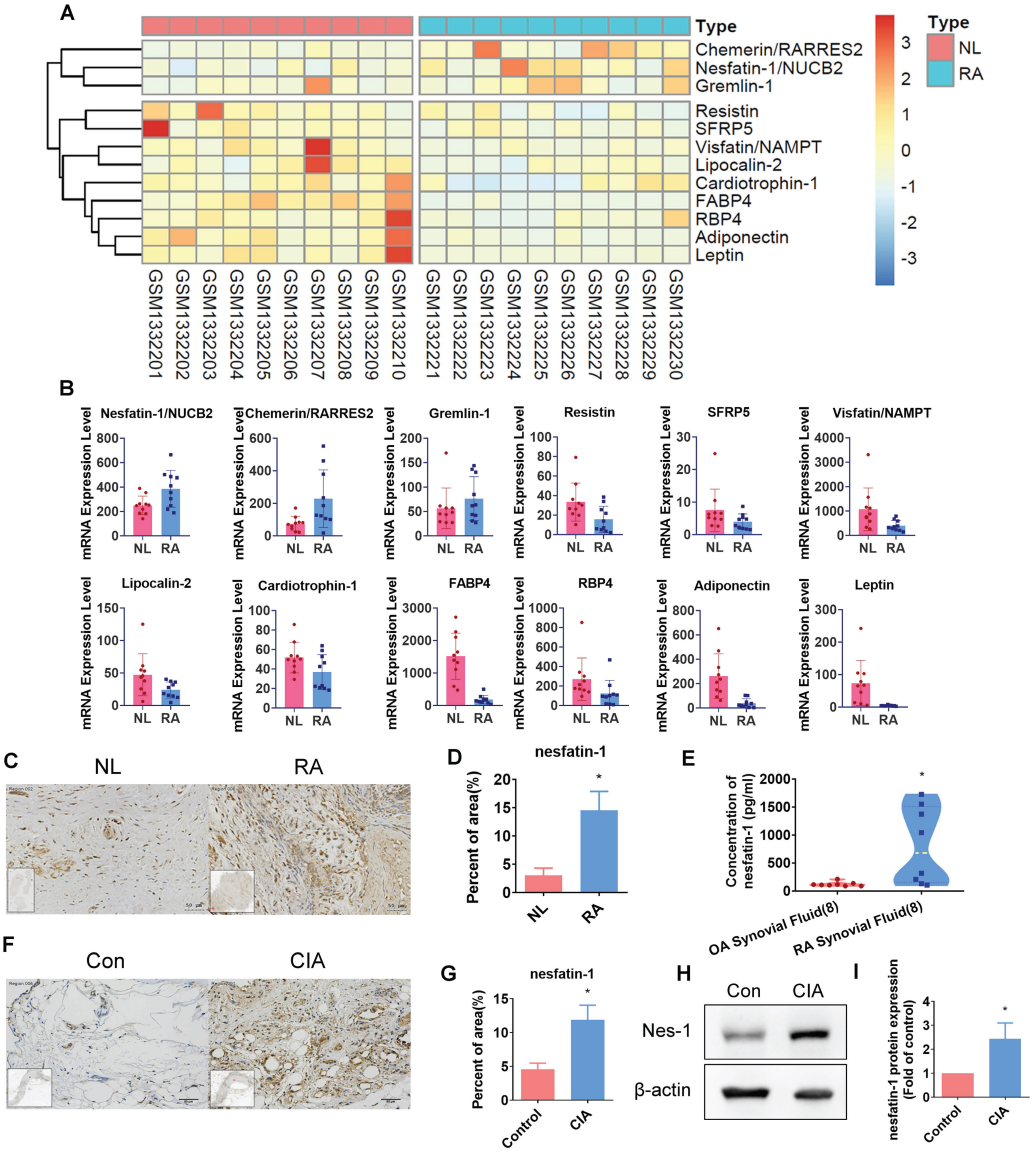
** Nesfatin-1 is the highly expressed adipokine observed in RA synovial tissue.** (A-B) Levels of nesfatin-1 and other adipokines in normal and RA synovial tissue samples obtained from the GEO dataset GSE55235. (C-D) IHC staining shows higher levels of nesfatin-1 in human RA synovial tissue (n = 4) compared with healthy tissue (n = 4). (E) ELISA analysis shows higher levels of nesfatin-1 in RA synovial fluid (n = 8) compared with OA synovial fluid (n = 8). (F-G) IHC staining shows CIA synovial tissue expressed higher levels of nesfatin-1 compared with controls (n = 3). (H-I) Western blot determined levels of nesfatin-1 expression in protein extracted from mouse forepaws (n = 3). * *p*<0.05 vs controls. NL: normal; RA: rheumatoid arthritis; OA: osteoarthritis; Con: control; CIA: collagen induced arthritis; Nes-1: nesfatin-1.

Our analysis of adipokines in the GEO dataset (GSE55235) revealed high levels expression of nesfatin-1 in RA synovial tissue compared with healthy donor samples (Figure 1A-B). Similarly, our IHC analysis of human RA and non-RA samples of synovial fluid and synovial tissue demonstrated significantly higher levels of nesfatin-1 expression in both synovial fluid and synovial tissue, compared with non-RA samples (Figure 1C-E). Moreover, nesfatin-1 expression levels were higher in CIA synovial tissue than in control tissue (Figure 1F-G); nesfatin-1 protein levels were also higher in CIA samples than in control samples (Figure 1H-I). Thus, GEO database records demonstrating high levels of nesfatin-1 expression in RA synovial tissue were also observed in our samples of human RA synovial tissue and in CIA synovial tissue.

### Nesfatin-1 increases CCL2 expression and facilitates THP-1 monocyte migration in RASFs

**Figure 2:**
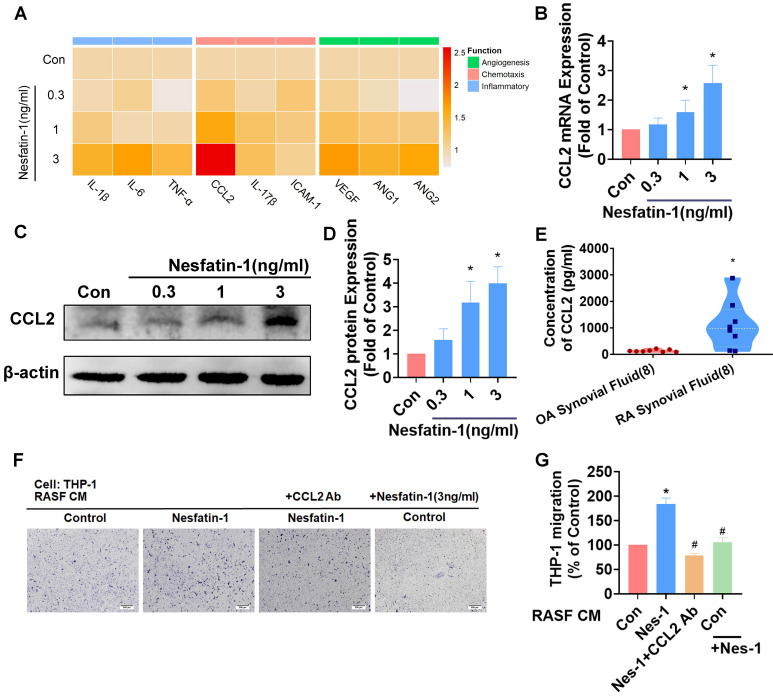
** Nesfatin-1 increases CCL2 expression in human RASFs and facilitates monocyte migration.** (A) RASFs were treated with nesfatin-1 (0.3-3 ng/mL) and the expression of 9 proinflammatory factors was examined by qPCR (n = 3). (B-D) RASFs were treated with nesfatin-1 (0.3-3 ng/mL) and levels of CCL2 expression were examined by qPCR (n = 6) and Western blot (n = 4). (E) ELISA analysis revealed higher synovial fluid levels of CCL2 among RA patients (n = 8) compared with OA patients (n = 8). (F-G) RASFs were incubated with nesfatin-1 for 24 h then stimulated with CCL2 antibody (1 μg/ml) for 30 min. The conditioned medium (CM) was stored up and treated to THP-1 cells, followed by measurement of THP-1 migration (n = 4). * *p*<0.05 vs controls; # *p*<0.05 vs the nesfatin-1-treated group.

Inflammatory cytokines and chemokines produced by synovial fibroblasts play a crucial role in the progression of RA disease^6^; the principal inflammatory cytokines are IL-1β, IL-6 and TNF-α, while chemokines CCL2, IL-17β and Intercellular Adhesion Molecule 1 (ICAM-1) regulate leukocyte migration and infiltration, and upregulated expression of angiogenic factors in RA include most notably vascular endothelial growth factor (VEGF), as well as angiopoietins -1 and -2 (Ang-1 and Ang-2). We examined messenger RNA (mRNA) expression for each of these 9 proinflammatory markers, by treating RASF cells with different concentrations of nesfatin-1 (0, 0.3, 1, 3 ng/ml) (Figure 2A). Nesfatin-1 dose-dependently increased *Ccl2* mRNA and protein levels, whereas only slight increases in mRNA expression were seen for the other cytokines (Figure 2A-D). CCL2 was highly expressed in RA synovial fluid (Figure 2E). Next, an *in vitro* chemotaxis assay was performed to confirm CCL2 function. CM from nesfatin-1-treated RASF cells increased THP-1 monocyte migration, which was abolished when the cells were pretreated with CCL2 neutralizing antibody, indicating that monocyte migration is controlled by nesfatin-1-mediated CCL2 expression (Figure 2F-G). Notably, nesfatin-1 treatment of RASF CM did not directly induce THP-1 monocyte migration (Figure 2 F-G). It appears that nesfatin-1 increases CCL2 expression and promotes monocyte migration in human RASFs.

### MEK/ERK and p38 signaling is involved in nesfatin-1-induced increases in CCL2 expression and monocyte migration in RASFs

**Figure 3:**
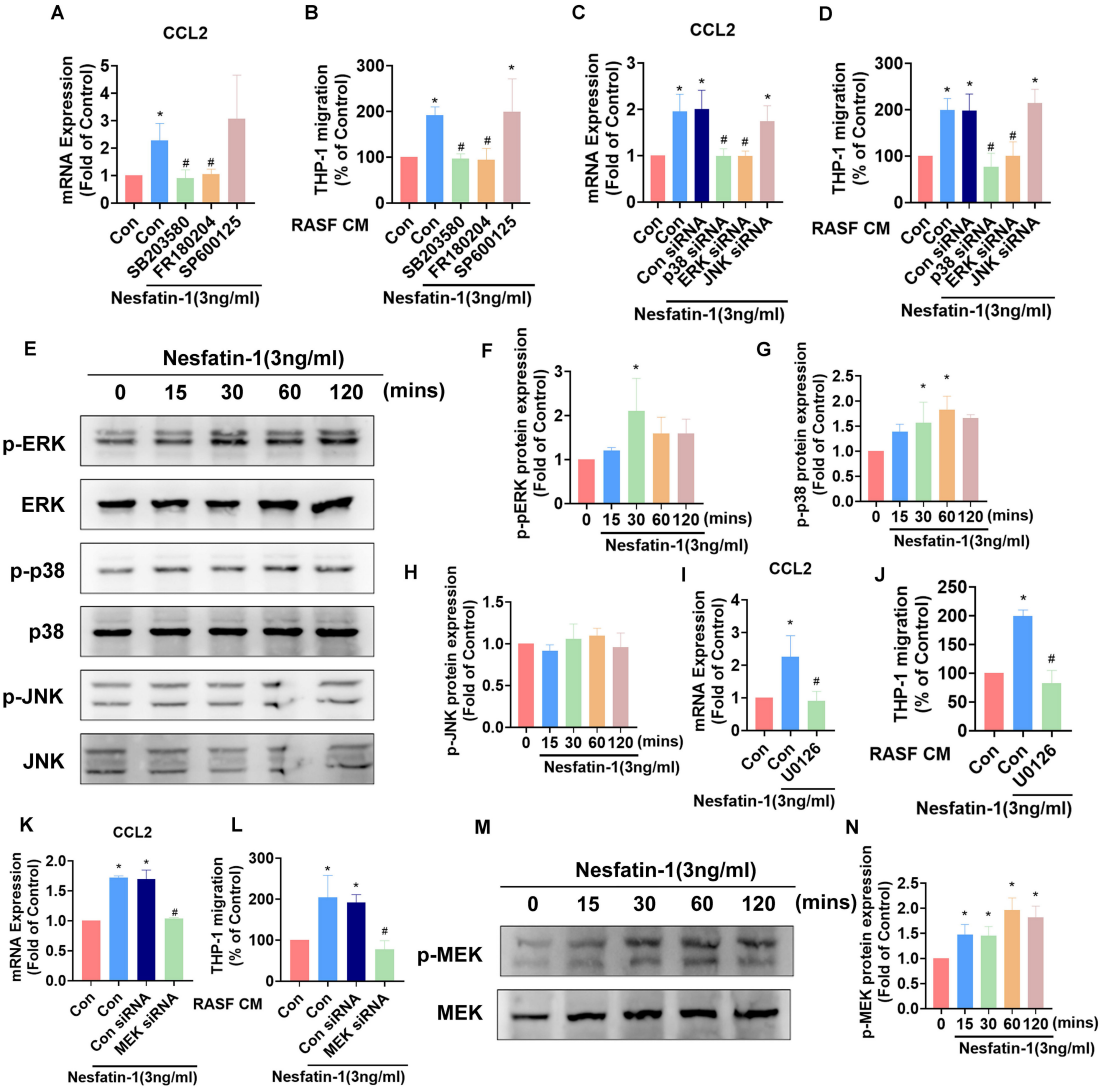
** The MEK/ERK and p38 pathways are involved in nesfatin-1-induced increases in CCL2 expression and monocyte migration.** (A-D) RASFs were treated with an ERK inhibitor (FR180204; 10μM), p38 inhibitor (SB203580; 10μM), or JNK inhibitor (SP600125; 10μM), or transfected with ERK, p38, and JNK siRNAs, then stimulated with nesfatin-1. CCL2 expression was analyzed by qPCR (n = 3) and CM was treated to THP-1 cells, followed by measurement of migration activity (n = 3). (E-H) RASFs were treated with nesfatin-1 for the designated time intervals; ERK, p38, and JNK phosphorylation was examined by Western blot (n = 3). (I-L) RASFs were treated with a MEK inhibitor (U0126; 10μM) or transfected with MEK siRNA then stimulated with nesfatin-1. CCL2 expression was examined by qPCR (n = 3) and CM was treated to THP-1 cells, followed by measurement of migration activity (n = 3). (M-N) RASFs were treated with nesfatin-1 for the designated time intervals; MEK phosphorylation was examined by Western blot (n = 3). * *p*<0.05 vs controls; # *p*<0.05 vs the nesfatin-1-treated group.

Mitogen-activated protein kinase (MAPK) signaling contributes to higher levels of CCL2 expression in inflammatory responses 28. Thus, we investigated how the MAPK cascade modulates nesfatin-1-induced increases in CCL2 expression. We observed that pretreating RASFs with either an ERK inhibitor (FR180204) or p38 inhibitor (SB203580) abolished the effects of nesfatin-1 upon levels of CCL2, whereas no such changes were observed when the cells were pretreated with a JNK inhibitor (SP600125) (Figure 3A). Pretreating RASFs with ERK and p38 inhibitors also abolished nesfatin-1-induced stimulation of monocyte migration (Figure 3B). Transfecting RASFs with ERK and p38 siRNAs reversed nesfatin-1-induced increases in CCL2 expression and monocyte migration (Figure 3C-D). Interestingly, nesfatin-1 treatment promoted ERK and p38 phosphorylation in a time-dependent manner, whereas JNK phosphorylation was not affected (Figure 3E-H). We then found that pretreating the cells with an ERK upstream MEK inhibitor (U0126) or MEK siRNA abolished nesfatin-1-induced increases in CCL2 expression and monocyte migration (Figure 3I-L), while treatment with nesfatin-1 promoted MEK phosphorylation (Figure 3M-N). These data suggest that nesfatin-1 induces increases in CCL2 expression via the MEK/ERK and p38 signaling pathways, facilitating monocyte migration.

### Nesfatin-1 upregulates CCL2 expression and promotes monocyte migration via NF-κB signaling in RASFs

**Figure 4:**
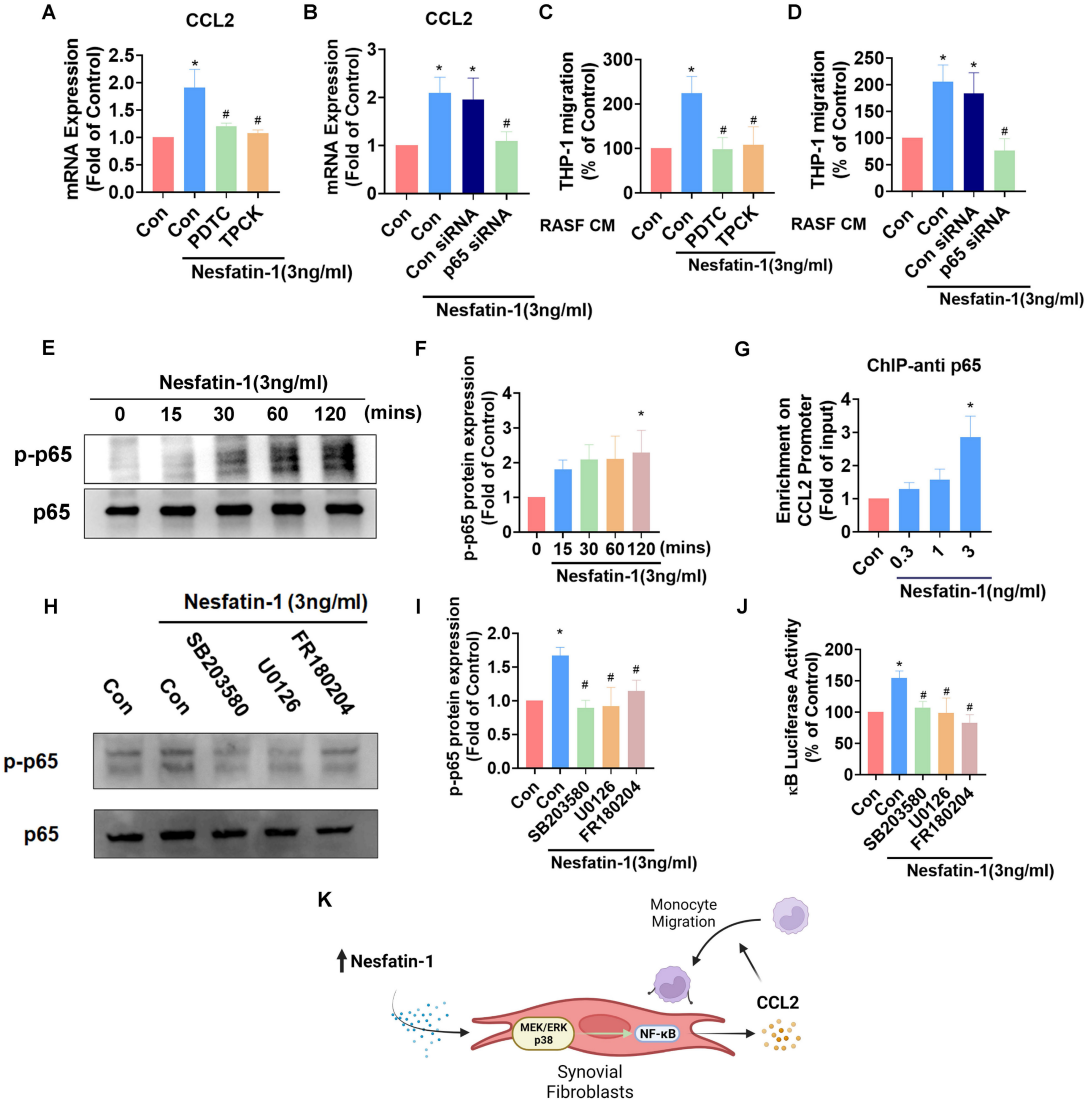
** The NF-κB pathway is involved in nesfatin-1-induced increases in CCL2 expression and monocyte migration.** (A-D) RASFs were treated with NF-κB inhibitors (PDTC; 3μM and TPCK; 1μM), or transfected with p65 siRNA, then stimulated with nesfatin-1. CCL2 expression was analyzed by qPCR (n = 4) and CM was treated to THP-1 cells, followed by measurement of migration activity (n = 4). (E-F) RASFs were treated with nesfatin-1 for the designated time intervals; p65 phosphorylation was examined by Western blot (n = 3). (G) RASFs were incubated with nesfatin-1 (0.3-3 ng/mL) and chromatin was immunoprecipitated with anti-p65 and examined by qPCR (n = 4). (H-I) RASFs were treated with the designated inhibitors then stimulated with nesfatin-1, and p65 phosphorylation was examined by Western blot (n = 3). (J) RASFs were treated with indicated inhibitors then stimulated with nesfatin-1, before examining NF-κB luciferase activity (n = 4). (K) Model showing how nesfatin-1 induces CCL2 expression and promotes monocyte migration in synovial fibroblasts via the MEK/ERK, p38 and NF-κB pathways. * *p*<0.05 vs controls; # *p*<0.05 vs the nesfatin-1-treated group.

NF-κB is a crucial transcription factor in the inflammatory response and a common downstream target of MAPK signaling 29. Incubating RASFs with NF-κB inhibitors (PDTC and TPCK) or transfecting the cells with p65 siRNA reduced nesfatin-1-induced upregulation of *Ccl2* mRNA expression (Figure 4A-B). These NF-κB inhibitors and p65 siRNA also decreased nesfatin-1-induced migration of THP-1 monocytes (Figure 4C-D). Nesfatin-1 treatment induced p65 phosphorylation in a time-dependent manner, while data from the ChIP assay revealed dose-dependent binding of p65 to the NF-κB element on the CCL2 promoter (Figure 4E-G). When we examined whether NF-κB is situated downstream of the MEK/ERK and p38 signaling pathways, pretreatment of RASFs with MEK/ERK and p38 inhibitors prevented nesfatin-1-induced increases in NF-κB luciferase activity and downregulated nesfatin-1-induced p65 phosphorylation (Figure 4H-J). These data suggest that nesfatin-1 upregulates CCL2 expression via the MEK/ERK, p38 and NF-κB signaling pathways in RASFs (Figure 4K).

### Nesfatin-1 upregulates CCL2 expression and enhances the polarization of THP-1-derived macrophages to the M1 phenotype

**Figure 5:**
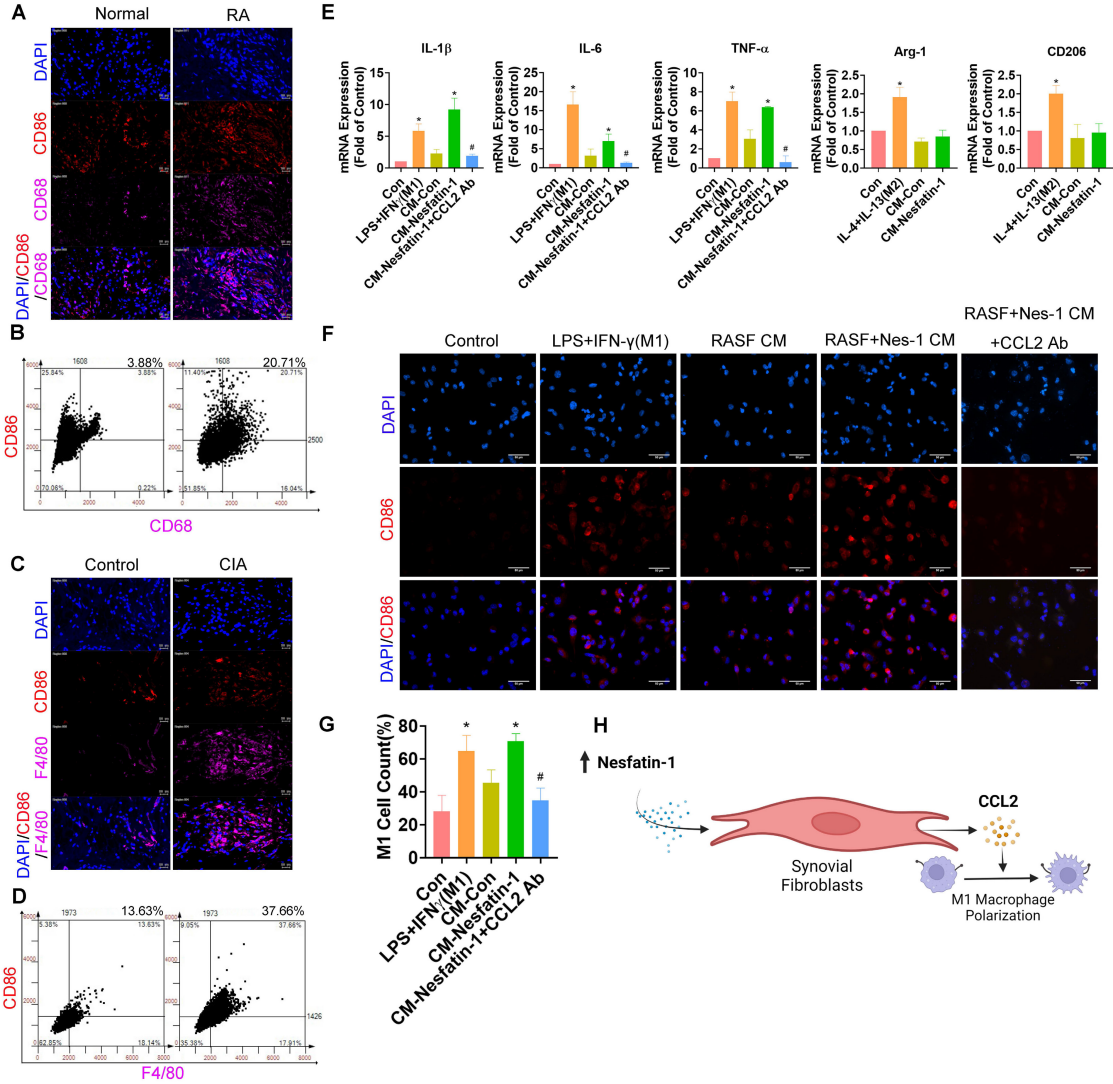
** Nesfatin-1-induced increases in CCL2 expression mediated M1 macrophage polarization.** (A) Immunofluorescence staining for CD68 and CD86 in synovium tissue from RA patients (n = 4) and healthy individuals (n = 4). (B) Quantification of F4/80 and CD86 expression from immunofluorescence staining. (C) Immunofluorescence staining for F4/80 and CD86 in synovium tissue from CIA mice (n = 6) and control mice (n = 6). (D) Quantification of CD68 and CD86 expression from immunofluorescence staining. (E-G) THP-1 cells were incubated with PMA for 24 h, then stimulated with LPS or CM for 24 h, before using qPCR (n = 3) to examine cytokine levels and immunofluorescence staining (n = 3) for CD86. (H) Model showing how nesfatin-1 induced increases in CCL2 expression in RASFs, which in turn facilitated monocyte migration and M1 macrophage polarization. * *p*<0.05 vs controls; # *p*<0.05 vs the nesfatin-1-treated group.

M1 macrophages play a crucial role in modulating the inflammatory response in RA synovial tissue 30. In our data, M1 macrophages were abundantly expressed in human RA synovial tissue but minimally in healthy samples, according to double immunofluorescence staining of the synovium with the macrophage marker CD68 and the M1 phenotype marker CD86 (Figure 5A-B). Similarly, M1 macrophage expression was higher in CIA synovial tissue than in samples from control mice (Figure 5C-D). We next investigated whether nesfatin-1-induced upregulation of CCL2 enhanced the polarization of M1 macrophages by treating THP-1-derived (M0) macrophages with synovial fibroblast CM. LPS + IFN-γ treatment and IL-4 + IL-13 treatment served as the positive controls of M1 and M2 macrophages, respectively. M0 macrophages incubated with nesfatin-1-treated RASF CM exhibited markedly greater polarization compared with M0 macrophages administered RASF CM only, according to levels of mRNA expression of M1 markers *IL-1β*, *IL-6* and *TNF-α* (Figure 5E). M1 macrophage polarization was promoted by nesfatin-1-induced increases in CCL2 expression and reduced by CCL2 neutralizing antibody (Figure 5E). Notably, incubating M0 macrophages with CM did not increase levels of M2 macrophage markers *Arg-1* and *CD206*, suggesting that nesfatin-1-induced increases in CCL2 expression does not affect M2 macrophage polarization (Figure 5E). These results were confirmed by immunofluorescence staining, showing increased expression of the M1 phenotype marker CD86. Similarly, treating M0 macrophages with synovial fibroblast CM increased CD86 expression (Figure 5F-G). These findings suggest that nesfatin-1-induced increases in CCL2 expression polarize naïve macrophages to the M1 phenotype (Figure 5H).

### Inhibiting nesfatin-1 ameliorates CIA severity

**Figure 6:**
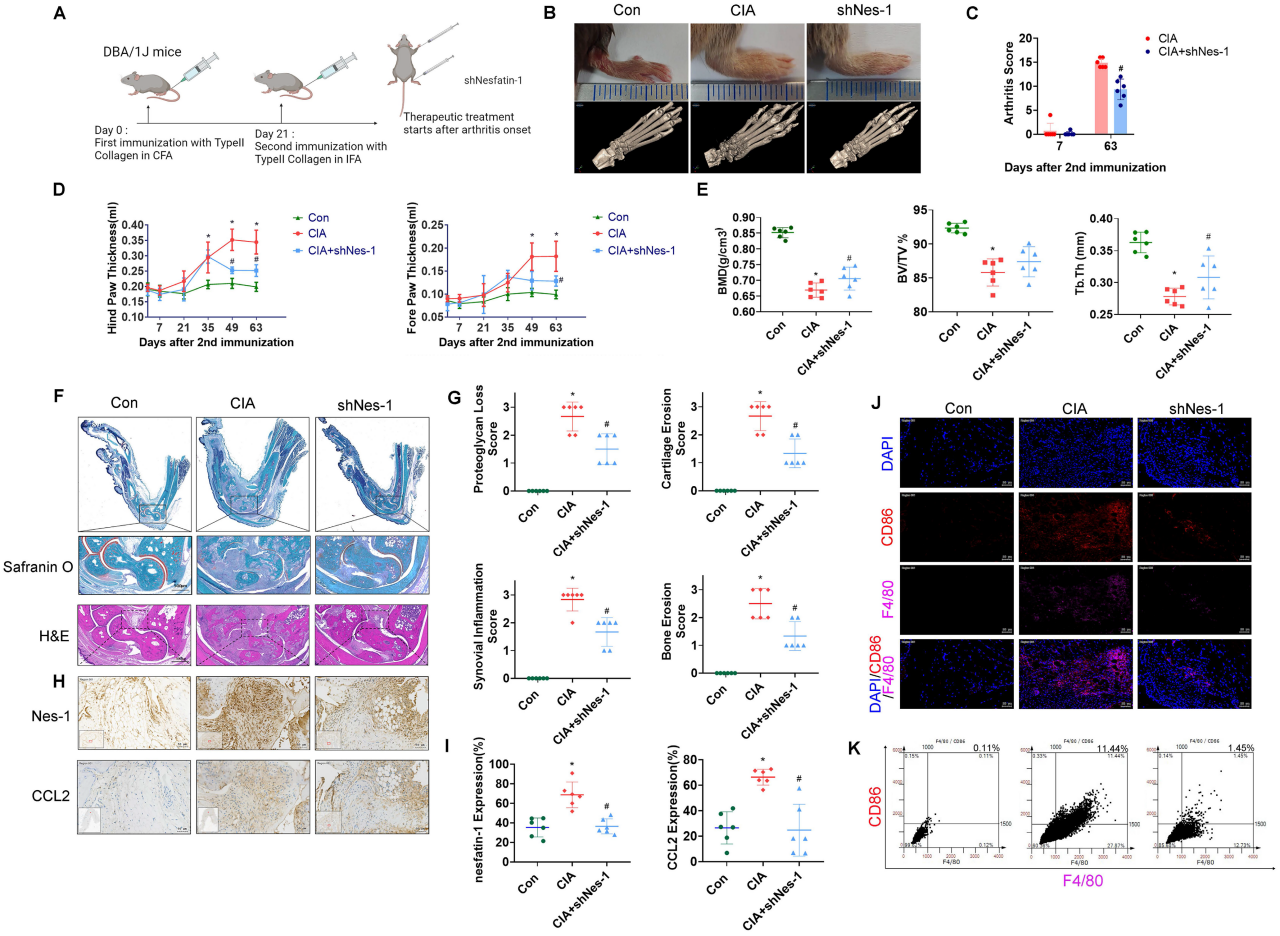
** Nesfatin-1 knockdown ameliorated paw swelling ,bone destruction, synovial inflammation and M1 macrophage expression in CIA mice.** CIA mice received intra-articular injections of nesfatin-1 shRNA on day 7, and were sacrificed on day 63 after the second immunisation (n = 6 mice for each group and time point). (A) CIA induction and nesfatin-1 shRNA injections workflow. (B) Hind paw swelling was photographed, and µ-CT images of hind paws were obtained on day 63 after the second immunisation. (C-D) Arthritis scores were monitored; hind paw and forepaw swelling was evaluated with a digital plethysmometer. (E) Quantification of BMD, BV/TV, and Tb.Th values (n = 6). (F) Representative images of histologic sections from ankle joints stained with Safranin-O/Fast Green and H&E. Dotted rectangle: synovial tissues from CIA mice. (G) Quantification of cartliage destruction, proteoglycan loss and inflammation scores in ankle joints (n = 6). (H-I) Representative images of histologic sections from ankle joints stained nesfatin-1 and CCL2 antibodies and quantification of nesfatin-1 and CCL2 expression (n = 6). (J-K) Immunofluorescence staining for F4/80 and CD86 in synovium tissue and quantification of F4/80 and CD86 expression (n = 6). * *p*<0.05 vs controls; # *p*<0.05 vs CIA mice.

**Figure 7:**
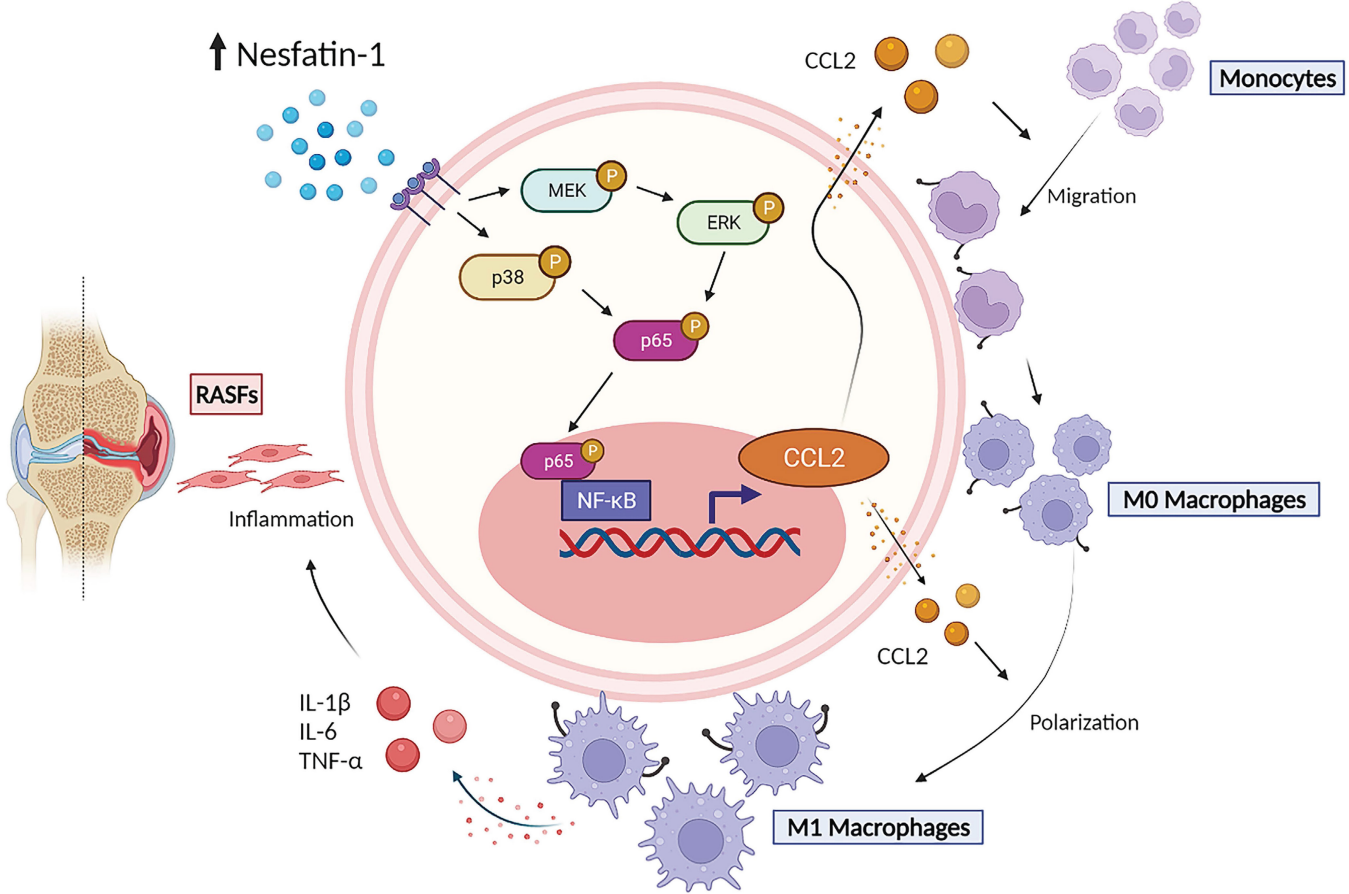
** The schematic diagram summarizes the role of nesfatin-1 in RA.** The schematic diagram summarizes the mechanisms that nesfatin-1 upregulates CCL2 production and thereby facilitates monocyte migration via the MEK/ERK, p38 and NF-κB signaling pathways, which promotes M1 macrophage polarization in human RASFs.

To examine the *in vivo* role of nesfatin-1, ankles and wrists of CIA mice were injected once weekly with nesfatin-1 shRNA for 8 weeks, before sacrificing the mice (Figure 6A). Nesfatin-1 shRNA was associated with significant reductions in swelling, arthritis scores, hind paw and forepaw thicknesses compared with CIA alone (Figure 6B-D). Analysis of the µ-CT images (Figure 6B) revealed that nesfatin-1 shRNA treatment ameliorated the bone destruction and decreases in bone mineral density (BMD), bone volume/tissue volume (BV/TV) and trabecular thickness (Tb.Th) observed in the CIA-only mice (Figure 6E). IHC findings showed significantly attenuated severity of cartilage destruction in mice treated with nesfatin-1 shRNA compared with mice in the CIA-only group, as well as markedly limited the infiltration of inflammatory cells into the synovium in the nesfatin-1 shRNA group compared with the CIA-only group (Figure 6F-G). Compared with CIA alone, nesfatin-1 shRNA distinctly reduced levels of nesfatin-1 and CCL2 protein (Figure 6H-I), while the immunofluorescence data revealed that nesfatin-1 shRNA significantly reduced the numbers of monocytes and thereby decreased the numbers of M1 macrophages (Figure 6J-K). It appears that knockdown of nesfatin-1 inhibits the severity of CIA disease. The schematic diagram highlights the ways in which nesfatin-1 can be considered as a diagnostic element and a therapeutic target in RA disease (Figure 7).

## Discussion

RA has long been recognized for its severe signs and symptoms of synovitis and joint destruction 2. The crucial role played by activated RASFs in RA disease pathology make these cells important immune/cytokine mediators 6. Increasing evidence attests to strong connections between adipokines and RA pathology 31, with for instance leptin, resistin, adiponectin, and visfatin influencing RA inflammation and disease progression, which have become important therapeutic targets in RA 32. In our analysis of the GEO database, nesfatin-1, chemerin, and gremlin-1 were highly expressed in RA synovial tissue compared with normal healthy tissue. Chemerin increases the production of IL-6, matrix metalloproteinase-3 (MMP-3) and levels of toll-like receptor 4 (TLR4) in RASFs 33, 34, while gremlin 1 promotes RASF hyperplasia, migration, and invasiveness 35. However, the effect of nesfatin-1 in RA is uncertain. This study reports that nesfatin-1-induced increases in synovial fibroblasts upregulated levels of CCL2, which is one of the most important regulatory chemokines of monocyte migration and infiltration in the inflammatory microenvironment. Interestingly, several adipokines have shown similar effects in joint diseases. For instance, resistin increases CCL2 expression in osteoarthritis (OA) synovial fibroblasts 36, while visfatin and adiponectin upregulate levels of several chemokines in OA and RA 37, 38. Thus, our evidence showing that nesfatin-1 induces increases in CCL2 expression provides a new insight into adipokine involvement in joint disease.

Controversy surrounds nesfatin-1 in arthritis diseases, as this adipokine exhibits both proinflammatory and anti-inflammatory functions. For example, nesfatin-1 can suppress IL-1β expression in OA chondrocytes and thus reduce the severity of cartilage destruction in OA rats 39, but nesfatin-1 can also facilitate IL-1β expression in OA synovial fibroblasts 40. Interestingly, nesfatin-1 shows protective effects in cartilage from rats with adjuvant-induced arthritis 41 and reportedly reduces the risk of atherosclerotic disease in patients with RA 42. Conversely, our study evidence reveals a proinflammatory effect of nesfatin-1 in RASFs and in CIA mice. Our study evidence suggests a new role for nesfatin-1 in RA disease.

The M1 macrophage phenotype is considered to be a crucial producer of proinflammatory cytokines IL-1β, IL-6 and TNF-α in RA synovium 30. In this study, levels of synovium M1 macrophages were significantly higher in RA patients and CIA mice compared with their healthy counterparts. This result is consistent with a previous report 43. Our study has also elucidated that nesfatin-1 induced increases in CCL2 in RASFs. Other research has found that CCL2 induces an M1-phenotype polarization at the transcriptomic and cellular functional levels in mouse and human macrophages 44, 45. We found that CM from nesfatin-1-treated RASFs increased the expression of M1 markers, but not M2 markers; CCL2 neutralizing antibody abolished the effect of nesfatin-1 treatment on M1 markers.

The MAPK signaling cascade is an important contributor to RA joint pathology 46 and, in particular, MAPK signaling upregulates CCL2 expression and worsens RA symptomatology 47. In this study, we found that nesfatin-1 stimulated MEK/ERK and p38 phosphorylation and thus upregulated CCL2 expression, but nesfatin-1 had no significant effect upon JNK phosphorylation. We also found that MEK/ERK and p38 inhibitors and siRNAs inhibited nesfatin-1-induced upregulation of CCL2 in RASFs. Our results showing a lack of effect of nesfatin-1 upon JNK phosphorylation despite significant stimulation of MEK/ERK and p38 phosphorylation are supported by another study, in which zearalenone enhanced the inflammatory response of mice testis through the activation of ERK and p38 signaling; JNK signaling was not involved 48. Our data also demonstrate that the nesfatin-1-induced increases in CCL2 production via MEK/ERK and p38 signaling stimulated RA monocyte migration.

Several studies have reported that the NF-κB signaling pathway upregulates many inflammatory cytokines 29, 49 and the activation of NF-κB signaling is known to increase CCL2 transcription and production 50. Our study found that NF-κB inhibitors TPCK and PDTC, as well as p65 siRNA, decreased nesfatin-1-induced increases in CCL2 expression and monocyte migration in human RASFs. The ChIP assay confirmed that p65 binds to the promoter site of the *Ccl2* gene and, furthermore, NF-κB luciferase activity was decreased by the MEK/ERK and p38 inhibitors, all of which reveals that nesfatin-1 upregulated NF-κB-dependent CCL2 expression and monocyte migration via MEK/ERK and p38 signaling.

Although we have discovered the mechanisms of nesfatin-1 induced increases CCL2 expression in RASF, the receptor of nesfatin-1 that mediates these effects has not been elucidated yet 51. Considering the effects of this receptor in many different diseases, this is thought to be a worthwhile target for future research. Moreover, although it is established that nesfatin-1 participates in human diseases, no published reports exist as yet regarding nesfatin-1 inhibitors. Designing effective nesfatin-1 inhibitors is an important topic for future research.

In summary, our study has demonstrated that nesfatin-1 is an important contributor to the disease process in RA synovium. We have provided evidence showing that increases in nesfatin-1 expression upregulated CCL2 expression in human RASFs and contributed to monocyte migration via the MEK/ERK, p38 and NF-κB signaling pathways. Furthermore, nesfatin-1-induced increases in CCL2 expression enhanced M1 polarization (Figure 7). Finally, nesfatin-1 effectively modulated RA severity in CIA mice. Our contention is that the study data support the use of nesfatin-1 as a diagnostic marker and as a therapeutic target in RA.

## Supplementary Material

Supplementary methods and tables.

## Abbreviations

RA: rheumatoid arthritis
OA: osteoarthritis
GEO: gene expression omnibus
IHC: immune histochemistry staining
CCL2: c-c motif chemokine ligand 2
IFN-γ: interferon gamma
IL-1β: interleukin 1 beta
IL-6: interleukin-6
TNF-α: tumor necrosis factor alpha
TGF-β: transforming growth factor beta
IL-10: interleukin-10
Arg-1: arginase 1
MAPK: mitogen-activated protein kinase
NF-κB: nuclear factor kappa B
SF: synovial fibroblast
qPCR: quantitative real-time polymerase chain reaction
ChIP: chromatin immunoprecipitation
CM: conditioned medium
BMD: bone mineral density
BV/TV: bone volume/tissue volume
Tb.Th: trabecular thickness
shRNA: short-hairpin RNA
