# Supplementary material for: Nesfatin-1 Stimulates CCL2-dependent Monocyte Migration And M1 Macrophage Polarization: Implications For Rheumatoid Arthritis Therapy
Source: Int J Biol Sci. 2023 Jan 1;19(1):281–93. doi: 10.7150/ijbs.77987 (PMC9760434; doi:10.7150/ijbs.77987)
Supplement: Supplementary file 1 — Supplementary methods and tables. [file ijbsv19p0281s1.pdf]

Supplementary Methods

GEO database analysis

A dataset of gene mRNA profiles in normal and RA synovial tissue (GSE55235) were downloaded from the GEO database and analyzed for adipokine expression.

Human clinical samples

Human synovial tissues (N=4) and synovial fluids (N=8) were collected from patients with RA/OA during total knee arthroplasty and from normal patients undergoing arthroscopy after joint/trauma derangement. This study approval was granted by the Institutional Review Board (IRB) of China Medical University Hospital, and all study patients completed written informed consent forms prior to study participation. All experiments involving human clinical samples were approved by the Institutional Review Board of China Medical University Hospital, which granted approval for this study to proceed (Approval no. CMUH108-REC3-039).

Collagen-induced arthritis (CIA) mouse model

DBA/1J mice (8–10 weeks old) were obtained from the National Laboratory Animal Center (Tainan, Taiwan). Bovine type II collagen (MD Bioproducts; 2 mg/ml) was dissolved in 0.05 M acetic acid and emulsified with an equal volume of Freund's complete adjuvant (Sigma-Aldrich). DBA/1J mice were immunized by intradermal injection at the base of the tail with 100 ml of the emulsion (day 0). Booster injections of 100 ml of emulsion consisting of 1:1 of Freund's incomplete adjuvant (Sigma-Aldrich) and type II collagen in 0.05 M acetic acid were injected at another site at the base of the tail on day 21[1]. After the second immunization, mice were randomly separated into different groups (Control, CIA control and CIA injected with nesfatin-1 shRNA, n=6 in each group). The mice received intra-articular injections containing ~7.1×10^6 plaque-forming units (PFU) of nesfatin-1 short hairpin RNA (shRNA) in the ankle every week for 8 weeks, starting on day 7 after the second immunization. Arthritis scores were monitored in a blinded fashion by researchers on day 7 and day 63 after the second immunization. Hind paw and forepaw swelling was evaluated every 2 weeks with a digital plethysmometer for 8 weeks. After sacrifice, ankle and phalange joints were obtained from each mouse and stored in 4% paraformaldehyde for analysis with micro-computed tomography (μ-CT) scanning. All animal experiments were conducted according to the guidelines and ethical policies by the China Medical University Institutional Animal Care and Use Committee (Approval no. CMUIACUC-2019-345-1).

Micro-Computed Tomography (μ-CT) Analysis

μ-CT scanning of the ankles was performed using an *in vivo* μ-CT scanner (Skyscan 1272; Bruker, Kontich, Belgium) at 8.0 mm resolution. The voltage was 60 kVp and the current was 280 μA at 5 watts micro-focus output. The region of interest (ROI) was defined as a bone area of a total 100 slices (1.8 mm) from the proximal junction of the calcaneus and navicular bone and extending into the tarsals in the mouse, as detailed in a previous report[2]. Reconstructed cross-sections were realigned and the ROI was selected for analysis using CTAn 1.20.8 software (Bruker micro-CT, Kontich, Belgium). We then used CtVox 3.3.0 software (Bruker micro-CT, Kontich, Belgium) for 3D visualization. Quantification of bone volume (BV), tissue volume (TV), trabecular thickness (Tb.Th), and bone mineral density (BMD) were defined in bone areas with manually drawn ROIs.

Supplementary Materials

Table S1. ELISA kit used in the present study

Gene	Species	Vendor	Catalog number
Nesfatin-1	H	R&D Systems	DY5949
CCL2	H	R&D Systems	DY279

Table S2. Antibodies used in the present study

Protein	Application	Vendor	Catalog number
β-Actin	WB	GeneTex	GT5512
Nesfatin-1	WB, IHC	NOVUS	NBP1-87383
CCL2	WB, Neutralization	R&D	MAB679
CCL2	IHC	R&D	AF-479
p-ERK	WB	Santa Cruz	SC-7383
ERK	WB	Santa Cruz	SC-1647
p-p38	WB	Santa Cruz	SC-166182
p38	WB	Santa Cruz	SC-7972
p-JNK	WB	Santa Cruz	SC-6254
JNK	WB	Santa Cruz	SC-7345
p-MEK	WB	Cell signaling	2338S
MEK	WB	Santa Cruz	SC-6250
p-p65	WB	Cell signaling	3033
p65	WB, ChIP	Santa Cruz	SC-8008
F4/80	IHC	Abcam	ab6640
CD68	IHC	GeneTex	GTX41865
CD86	IHC	ABclonal	A1199

Table S3. Sequences of RT-PCR primers

Gene	Species	Forward primer	Reverse primer
β-Actin	H	CATGTACGTTGCTATCCAGGC	CTCCTTAATGTCACGCACGAT
Arg-1	H	GGTTTTTGTTGTTGCGGTGTTC	CTGGGATACTGATGGTGGGATGT
ANG1	H	AGCGCCGAAGTCCAGAAAAC	TACTCTCACGACAGTTGCCAT
ANG2	H	AACTTTCGGAAGAGCATGGAC	CGAGTCATCGTATTCGAGCGG
CCL2	H	CAGCCAGATGCAATCAATGCC	TGGAATCCTGAACCCACTTCT
CD206	H	GGGTTGCTATCACTCTCTATGC	TTTCTTGTCTGTTGCCGTAGTT
CD86	H	CCATCAGCTTGTCTGTTTCATTCC	GCTGTAATCCAAGGAATGTGGTC
ICAM-1	H	ATGCCCAGACATCTGTGTC	GGGGTCTCTATGCCCAACAA
IL-1β	H	ATGATGGCTTATTACAGTGGCAA	GTCGGAGATTCGTAGCTGGA
IL-6	H	AGACAGCCACTCACCTCTTCAG	TTCTGCCAGTGCCTCTTTGCTG
IL-17β	H	GCTGTGGATGTCCAACAAGAGG	TCCTGCATGGTGAAGGGGTTCA

TNF-α	H	CCTCTCTCTAATCAGCCCTCTG	GAGGACCTGGGAGTAGATGAG
VEGF	H	AGGGCAGAATCATCACGAAGT	AGGGTCTCGATTGGATGGCA
CCL2 promoter	H	CCTGGAAATCCACAGGATGC	CGAGAGTGCGAGCTTCAG

Table S4. Inhibitors used in the present study

Gene	Name	Working concentration	Vendor	Catalog number
p38	SB203580	10 μM	ENZO	BML-EI286-0001
ERK	FR180204	10 μM	Santa	SC-203945
JNK	SP600125	10 μM	ENZO	BML-EI305-0010
MEK	U0126	10 μM	Sigma	U120-1MG
κB	PDTC	3 μM	Sigma	P8765-1G
κB	TPCK	1 μM	Sigma	T4376-100MG

Table S5. siRNA used in the present study

Gene	Species	Vendor	Catalog number
p38	H	Dharmacon	L00351200
ERK	H	Dharmacon	L00355500
JNK	H	Dharmacon	L00351400
MEK	H	Dharmacon	L00357100
p65	H	Dharmacon	L-003533-00-0005
Control	H	Dharmacon	D-001810-10-05

Reference:

1. Achudhan D, Liu S-C, Lin Y-Y, Huang C-C, Tsai C-H, Ko C-Y, et al. Antcin K Inhibits TNF-α, IL-1β and IL-8 Expression in Synovial Fibroblasts and Ameliorates Cartilage Degradation: Implications for the Treatment of Rheumatoid Arthritis. Frontiers in Immunology. 2021; 12.
2. Lord AE, Zhang L, Erickson JE, Bryant S, Nelson CM, Gaudette SM, et al. Quantitative in vivo micro-computed tomography for monitoring disease activity and treatment response in a collagen-induced arthritis mouse model. Sci Rep. 2022; 12: 2863.
